# Rapidly Progressive Cerebellar Syndrome Associated With Anti-Yo Antibodies Following Complete Response to Advanced Ovarian Carcinoma Treated With Olaparib: A Case Report

**DOI:** 10.7759/cureus.99473

**Published:** 2025-12-17

**Authors:** Sara Carvalho, Ana Carolina Lopes, Ana Rita Lopes

**Affiliations:** 1 Medical Oncology, Instituto Português de Oncologia do Porto Francisco Gentil (IPO Porto), Porto, PRT; 2 Neurology, Instituto Português de Oncologia do Porto Francisco Gentil (IPO Porto), Porto, PRT

**Keywords:** advanced ovarian cancer, anti-yo antibodies, paraneoplastic neurological syndrome, parp inhibitor therapy, recurrence predictors

## Abstract

Rapidly progressive cerebellar syndrome (RPCS) mediated by anti-Yo antibodies is a rare neurological manifestation of paraneoplastic syndromes, most often associated with active breast or gynecological neoplasms. Its occurrence after apparent remission of cancer is rarely described and carries a poor prognosis.

We report the case of a 47-year-old woman with stage IIIC high-grade serous ovarian carcinoma (International Federation of Gynecology and Obstetrics (FIGO)) treated with neoadjuvant chemotherapy, complete cytoreductive surgery, and maintenance treatment with olaparib. Two weeks after starting the PARP inhibitor (iPARP), she developed nausea, vomiting, and vertigo, initially attributed to drug toxicity. The dose was reduced by one level, with improvement in gastrointestinal complaints. Months later, she progressed with ataxia, dysarthria, and postural instability. An exhaustive neurological study was performed, which revealed positive anti-Yo and anti-amphiphysin antibodies. Imaging and tumor markers showed no evidence of disease recurrence. A diagnosis of paraneoplastic RPCS was made. Sequential treatment with corticosteroids, intravenous immunoglobulin, and cyclophosphamide was ineffective. It was only about six months after the diagnosis of RPCS that a locoregional recurrence was identified. The patient started carboplatin in monotherapy due to clinical frailty, with improvement in speech impairment after only one cycle.

This case highlights the diagnostic complexity in distinguishing between drug-related toxicity and immune-mediated neurological syndromes. Although there is no direct evidence linking olaparib to the induction of paraneoplastic syndromes, its immunomodulatory effects may unmask latent autoimmune responses in susceptible individuals. Anti-Yo RPCS remains a therapeutic challenge with a poor neurological prognosis. Early recognition is crucial, and further research is needed to understand the interactions between targeted therapies and paraneoplastic immunity.

## Introduction

Paraneoplastic rapidly progressive cerebellar syndrome (RPCS) associated with anti-Yo antibodies is classically described in breast or gynecological neoplasms, and is more frequent when the oncological disease is active. Before establishing the diagnosis, one must exclude neurological impairment resulting from direct tumor invasion (e.g., brain metastases, carcinomatous meningitis) or secondary mechanisms such as coagulation disorders, therapy-related toxicity, metabolic alterations, or infections [1,2]. In addition to their potential for direct toxicity, some systemic treatments, including PARP inhibitors (iPARPs), have also been described as possible facilitators of immune responses [3]. Its occurrence in the absence of active disease is extremely rare and is usually associated with a worse prognosis, and only a few isolated cases have been documented in the literature [4].

The pathophysiology involved is still unclear, but it is presumed to be mediated by immunological phenomena, with the presence of antibodies being one of the data supporting this hypothesis [5]. According to the literature, RPCS is most often synchronous with or precedes the diagnosis of neoplasia [6].

Here, we report a case of RPCS in a survivor of ovarian carcinoma, the first clinical manifestation of the disease approximately two months after curative treatment and coinciding with the start of maintenance treatment with olaparib. This case was described according to the CARE (CAse REport) guidelines [7].

## Case presentation

**Figure 1:**
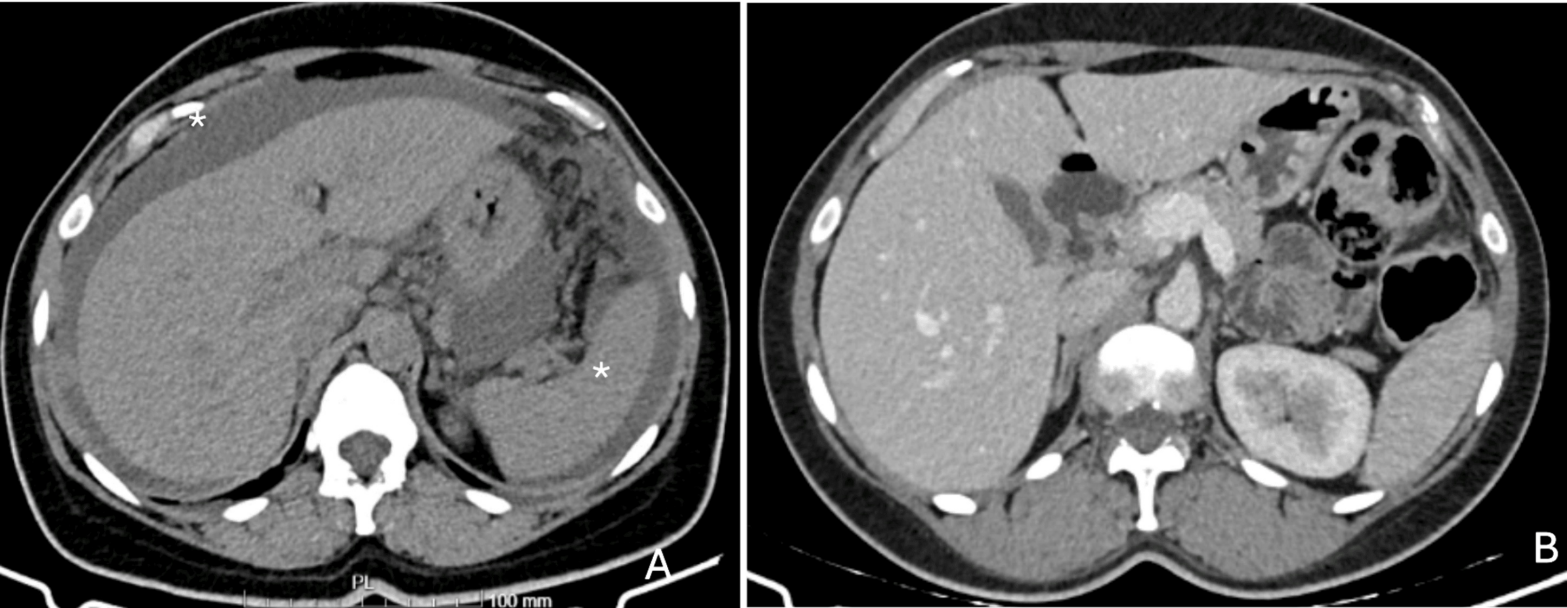
CT-TAP at diagnosis (A) and after complete cytoreductive surgery (B) (A) Axial contrast-enhanced thoracoabdominal CT at diagnosis demonstrating large-volume ascites (*), and extensive peritoneal involvement, with free fluid surrounding the liver and spleen. (B) Axial postoperative CT-TAP following complete cytoreductive surgery, showing resolution of ascites and no radiological evidence of residual peritoneal disease, consistent with a complete radiologic response. CT-TAP: computed tomography of the thorax, abdomen, and pelvis

The patient is a 47-year-old woman with no significant comorbidities and a history of a single episode of peripheral vertigo in October 2022, with no functional impact. She presented to the emergency department in October 2023 with progressive abdominal pain that had been worsening since May of that year. Physical examination revealed a painful pelvic mass, ascites, and suspected peritoneal carcinomatosis. A computed tomography of the thorax, abdomen, and pelvis (CT-TAP) confirmed large-volume ascites (Figure 1A), suspected peritoneal carcinomatosis, and two confluent neoformative lesions measuring 13.3 x 8.8 cm, involving the sigmoid colon and the posterior wall of the uterus. She also had lesions that bulged the hepatic margin, with no parenchymal lesions described. Her cancer antigen 125 (CA-125) tumour marker was 1098 U/mL. The biopsy confirmed high-grade serous carcinoma of the ovary (HGSOC), staged as IIIC according to the International Federation of Gynecology and Obstetrics (FIGO) 2014 classification. It was decided to start primary chemotherapy with carboplatin/paclitaxel, which began in December 2023.

After three cycles, she responded favourably to chemotherapy but still had unresectable disease, and it was decided to continue systemic treatment, completing a total of seven cycles in May 2024, with a favourable response, thus allowing, after multidisciplinary discussion, to propose maximum effort surgery.

She underwent complete cytoreductive surgery, which included total hysterectomy with bilateral adnexectomy, resection of the peritoneum from the bottom of the Douglas pouch and vesicouterine, plus resection of carcinomatosis lesions and omentectomy in August, with no macroscopic residual disease. The histological specimen confirmed HGSOC (CRS - Chemotherapy Response Score of 2), with an associated pathogenic variant of the somatic BRCA1 gene, not present in the germ line. Postoperative CT-TAP showed no evidence of disease (Figure 1B).

She started maintenance olaparib on 04 October 2024, presenting approximately two weeks later with complaints of nausea, vomiting, and vertigo aggravated by head rotation. A head CT-scan was performed, which showed no structural or vascular changes. Due to the possibility of iatrogenesis associated with olaparib, it was decided to reduce the dose, which improved the gastrointestinal symptoms, but the vertigo persisted.

On 04 February 2025, the patient presented to the emergency department with recurrent diplopia, dizziness, and worsening vertigo associated with gait instability. She reported that these symptoms had begun after initiating olaparib, with progressive worsening since January. Ophthalmologic evaluation excluded an ocular cause, while the otorhinolaryngology assessment ruled out a peripheral vestibular etiology. Neurological examination revealed mild dysarthria, horizontal gaze-evoked diplopia, left limb dysmetria, and marked postural instability with deviation to the left - findings consistent with a left cerebellar syndrome.

Hospitalization was decided upon to continue monitoring and studying. Brain MRI was unremarkable, showing no structural abnormalities to account for the symptoms. Cerebrospinal fluid (CSF) analysis revealed no significant cytochemical, microbiological, or cytopathological alterations. Anti-yo and anti-amphiphysin antineuronal antibodies were positive. Considering the serological and clinical findings, a diagnosis of paraneoplastic RPCS was made (Table 1).

**Table 1:** Laboratory findings Results in favor of paraneoplastic rapidly progressive cerebellar syndrome (RPCS). CSF: cerebrospinal fluid; PCR: polymerase chain reaction; WBC: white blood cells

Analysis	CSF	Reference values
Glucose	65 mg/dL	50-79 mg/dL
Proteins	56 mg/dL	15-45 mg/dL
Bacteriology and PCR for the virus	Undetected	Negative
CSF cytology	No malignant cells identified. White Blood Cells (WBC) - 4 nucleated cells/µL	WBC 0-5/µL
Antineuronal antibodies	Positive for anti-YO and anti-anfifisina; negative for anti-Ri, anti-Hu, anti-CV2, anti- Ma2/TA, anti-Tr, anti-GAD65, anti-Zic4, and anti-SOX1	Negative

**Figure 2:**
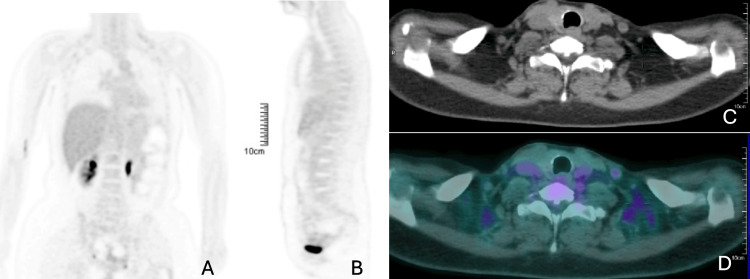
Whole-body PET/CT at the time of neurological symptom onset showing no evidence of metabolically active disease (February 2025) (A) Coronal FDG-PET image demonstrating physiological tracer distribution without abnormal hypermetabolic foci in the thorax, abdomen, or pelvis. (B) Sagittal FDG-PET image likewise showing absence of pathological FDG uptake. (C) Corresponding axial CT image at the cervicothoracic level showing no structural abnormalities or suspicious soft-tissue lesions. (D) Axial fused PET/CT image of the same level, confirming the absence of abnormal FDG uptake and supporting the lack of metabolically active disease. PET/CT: positron emission tomography/computed tomography; FDG: fluorodeoxyglucose

At this point, a new positron emission tomography (PET) scan was performed, which showed no evidence of cancer recurrence (Figure 2).

High-dose corticosteroid therapy was initiated with intravenous methylprednisolone (1 g/day for five days), with minimal clinical improvement. A subsequent five-day course of intravenous immunoglobulin (IVIG, 1 g/day) was administered, also without benefit.

Due to the lack of response to first-line immunotherapy, monthly cyclophosphamide therapy was initiated in April 2025, with a total of three cycles completed by the end of June. Olaparib was discontinued during cyclophosphamide administration because of the potential for cumulative hematologic toxicity. At the time of discontinuation, no additional interactions or immune-related concerns were considered. No neurological improvement was observed, and further deterioration ensued; the patient decided not to continue cyclophosphamide therapy thereafter.

Other treatments, such as plasmapheresis and rituximab, were not considered because the patient was already in a fragile clinical condition and reluctant to undergo further immunosuppressive treatment with an uncertain likelihood of benefit.

**Figure 3:**
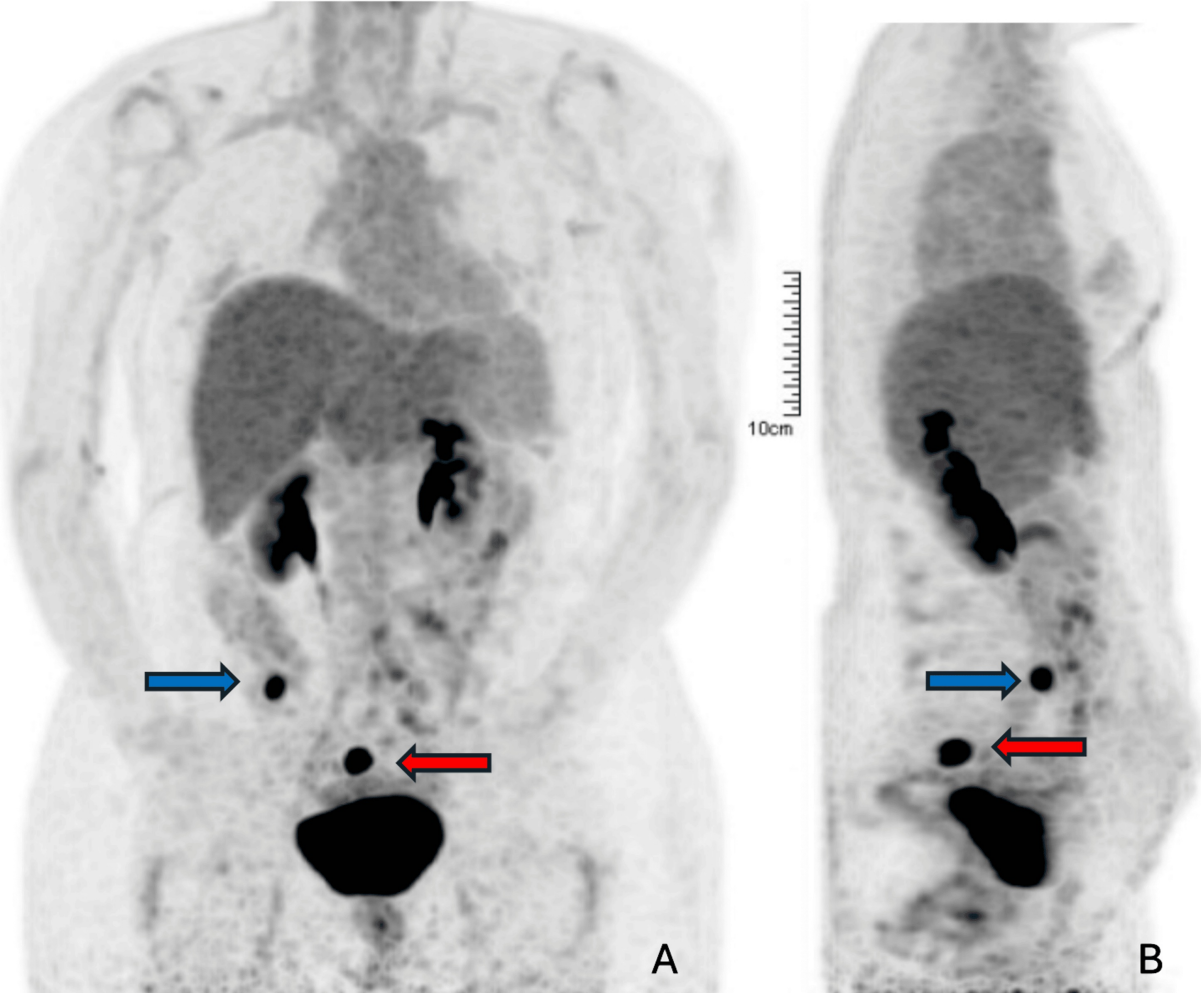
PET-scan showing the first radiologic evidence of disease recurrence, detected six months after neurological symptoms began (August 2025) (A) Coronal FDG-PET image showing a focal hypermetabolic area in the right iliac fossa (blue arrow; SUVmax 18.27) and a second hypermetabolic focus in the presacral region (red arrow; SUVmax 19.39), both adjacent to intestinal loops. (B) Sagittal FDG-PET view confirming the same hypermetabolic foci (blue and red arrows), persistent and with increased uptake on delayed imaging, findings suspicious for metastatic peritoneal implants. PET: positron emission tomography; FDG: fluorodeoxyglucose; SUVmax: maximum standardized uptake value

Throughout the treatments, serial imaging (February and May 2025) and laboratory assessments were performed, showing no evidence of oncological recurrence until August. On 26 August 2025, a PET scan was performed and revealed two newly developed pelvic peritoneal soft-tissue nodules - right iliac fossa (maximum standardized uptake value (SUVmax) 18.27) and presacral region (SUVmax 19.39) - suggestive of metastases. The tumour marker remained within normal limits (Figure 3).

**Figure 4:**
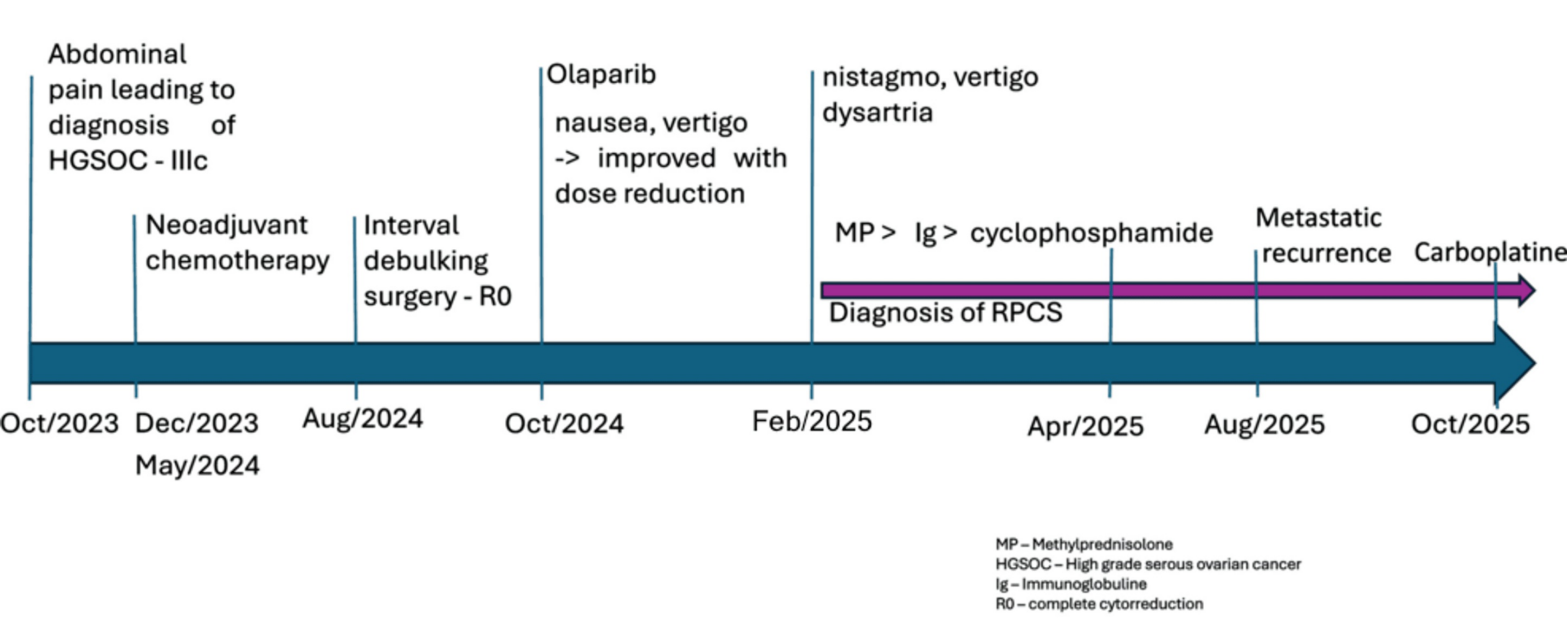
Timeline illustrating the clinical course, oncologic treatments, and neurological presentation It depicts the sequence from the diagnosis of high-grade serous ovarian carcinoma (HGSOC IIIc), neoadjuvant therapy, interval debulking surgery, initiation of olaparib, onset of cerebellar symptoms leading to the diagnosis of rapidly progressive cerebellar syndrome (RPCS) and immunomodulatory treatment, to metastatic recurrence and re-treatment with carboplatin (October 2023-October 2025).

A biopsy was performed, confirming recurrence of HGSOC. On 21 October 2025, the patient started carboplatin in monotherapy due to clinical frailty related to her neurological symptoms, with improvement in speech impairment after only one cycle, and currently the patient is still undergoing systemic treatment (Figure 4).

Clinically, to date, she continues to experience disabling vertigo, imbalance, and complete loss of independent ambulation, requiring institutional rehabilitation care. Higher cognitive functions remain unchanged. More time is needed to evaluate the response to the chemotherapy regimen, and although low, there is a chance of improvement.

## Discussion

The initial symptoms presented by the patient (vertigo, nausea, and vomiting) appeared approximately two weeks after the start of maintenance therapy with olaparib, which initially raised suspicion of a possible adverse effect. As a possible confounder, the patient had a previous history of vertigo syndrome. These symptoms are common in the first days or weeks of using iPARPs [8,9]. It is difficult to conclude whether this temporal overlap may have led to a delay in the diagnosis of cerebellar syndrome, since the neurological symptoms were consistent with the expected toxicity of the drug, and there was improvement in nausea with dose reduction. However, the persistence and progression of the remaining symptoms, with the appearance of objective cerebellar signs (axial ataxia, dysarthria, nystagmus), in a more complex clinical pattern inconsistent with exclusive pharmacological toxicity, was fundamental in raising the hypothesis of an immune-mediated neurological process, later confirmed by the positivity of anti-Yo antibodies. Concurrent positivity for anti-Yo and anti-amphiphysin antibodies is uncommon, as most published studies describe anti-Yo reactivity alone in cases of paraneoplastic cerebellar degeneration [2,4]. The prognostic significance of dual-antibody patterns remains unclear.

In retrospect, a referral to a neurologist might have been considered earlier, once symptoms persisted despite dose adjustment. However, the initial clinical presentation was indistinguishable from the expected treatment-related effects. This highlights how timely specialist input can be crucial in distinguishing drug toxicity from emerging paraneoplastic phenomena in similar scenarios.

This case highlights the need for rigorous clinical surveillance and a low threshold of suspicion to proceed with further diagnostics in patients with persistent or atypical central nervous system symptoms, even without evidence of active cancer and/or under targeted maintenance therapy.

Although there is still no direct evidence that olaparib acts as a facilitator of paraneoplastic neurological syndromes, there is a hypothesis that it may modulate the immune response, potentially favouring the exacerbation of pre-existing autoimmune responses [3,10]. Thus, the present case highlights not only the diagnostic difficulty inherent in the overlap of pharmacological toxicity and immune-mediated neurological disease but also raises the need for further studies on possible facilitating mechanisms between targeted therapies and paraneoplastic immunity.

Regarding our patient's RPCS, it evolved as described in the literature, with refractoriness to the treatments instituted. Even in the absence of confirmation of oncological recurrence, its prognosis is guarded and may be the first sign of a possible relapse, preceding radiological and biochemical confirmation [11], as was the case here.

There are still no recommendations on the best immunosuppressive strategy to offer, but it may include corticosteroids, immunoglobulins, plasmapheresis, cyclophosphamide, azathioprine, and rituximab [12]. Although rare, there are reported cases of long-term survivors with mild symptoms controlled pharmacologically [13]. It is unclear whether additional immunosuppressive strategies, such as plasmapheresis or rituximab, would have altered the clinical trajectory. Evidence of their efficacy in this setting is limited, and the patient refused them. In addition, cancer treatment should be offered if the cancer is confirmed.

With the current evidence of recurrence, it was not necessary to weigh the risks/benefits of reintroducing iPARP; a new platinum-based treatment was proposed, which may result in an improvement in the neurological syndrome itself, although this is unlikely.

## Conclusions

Anti-Yo paraneoplastic cerebellar syndrome should be considered in the differential diagnosis of neurological symptoms in cancer patients, even in the context of complete remission and under maintenance therapy. The lack of response to immunosuppressive therapies and the poor prognosis illustrate the severity and refractoriness typical of this syndrome, whose manifestation may precede confirmation of cancer relapse, as demonstrated in this case.

This case highlights the need for active surveillance for cancer recurrence when a paraneoplastic syndrome is present, as recurrence may occur late and oncologic therapy may be the only effective intervention. We also highlight the need for prolonged neurological surveillance and future research on potential interactions between targeted therapies and paraneoplastic autoimmune phenotypes.
